# Validating the Adult Concentration Inventory for Measuring Cognitive Disengagement Syndrome in an Iranian Population: Insights Into Mental Health and Cognitive Functioning

**DOI:** 10.1002/brb3.71033

**Published:** 2025-11-10

**Authors:** Karim Abdolmohamadi, Asgar Alimohamadi

**Affiliations:** ^1^ Department of Psychology Azarbaijan Shahid Madani University Tabriz Iran; ^2^ Faculty of Psychology and Education Allameh Tabataba'i University Tehran Iran

**Keywords:** Adult Concentration Inventory, cognitive disengagement syndrome, Iran, psychometrics

## Abstract

**Background:**

Recently, research on cognitive disengagement syndrome (CDS)—previously known as sluggish cognitive tempo—has expanded considerably. The Adult Concentration Inventory (ACI) is designed to assess CDS, and its psychometric soundness, including validity and reliability, needs to be evaluated across diverse countries and populations.

**Methods:**

This study assesses the questionnaire's psychometric properties in Iran. The study included 2855 people, 69% of whom were female and had a mean age of 27.42. The ACI questionnaire, Patient Health Questionnaire‐9 (PHQ‐9), and Executive Skills Questionnaire‐Revised (ESQ‐R) were completed in this study.

**Results:**

We found that the three‐factor model (attention problems, sluggishness, and mind‐wandering) matches the questionnaire using exploratory and confirmatory analysis. Based on the fit indices, the original correlated three‐factor model of the ACI was considered adequate: *χ* ^2^ = 815.20; df = 101; RMSEA = 0.050; CFI = 0.95; TLI = 0.94; IFI = 0.95; SRMR = 0.03.

**Conclusions:**

The results of this study demonstrated that CDS in Iranian society may be measured using the ACI questionnaire.

## Graphical abstract

This study validated the Adult Concentration Inventory (ACI) for assessing Cognitive Disengagement Syndrome (CDS) in an Iranian population. Data were collected from 2,855 adults (69% female; mean age = 27.42 years). Using the ACI, PHQ‐9, ESQ‐R, and Barkley Adult ADHD Rating Scale‐IV, a three‐factor structure emerged—Attention Difficulties, Slowness, and Daydreaming. The ACI demonstrated strong convergent validity with depression, slow cognitive tempo, and executive dysfunction, as well as satisfactory divergent validity based on the Fornell‐Larcker test. Overall, the findings confirmed that the ACI is a valid and reliable tool for assessing CDS among Iranian adults.

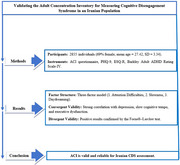

## 1 Introduction

Over the last two decades, there has been a growing number of research efforts centered around cognitive disengagement syndrome (CDS), which was formerly referred to as sluggish cognitive tempo (SCT) (Becker et al. 2023). This syndrome is categorized as one related to attention deficits (East et al. 2023) encompasses symptoms like drowsiness, daydreaming, lethargy, behavioral and cognitive sluggishness, and mental disorientation (Fredrick and Becker 2023). Studies have demonstrated a correlation between elevated symptoms of CDS and deficits in general, social, academic, and vocational functioning (Becker and Willcutt 2019; Becker et al. 2023).

### 1.1 Theoretical Framework

CDS was first utilized as a distinct subset of symptoms associated with attention deficit/hyperactivity disorder (ADHD) (Wang et al. 2025). Nonetheless, Studies on children with CDS have shown that the syndrome and ADHD are independent yet interconnected. Moreover, while the etiology of ADHD is predominantly genetic, preliminary studies indicate that the symptoms of CDS exhibit lower heritability and may be affected by environmental factors, including psychosocial adversity (Fredrick et al. 2019; Musicaro et al. 2020) or insufficient sleep (Becker, Epstein et al. 2019). Specifically, Symptoms of elevated CDS in adults are correlated with: (1) diminished executive functioning in daily activities and increased functional impairment in specific academic, occupational, financial, domestic, social, and interpersonal domains (Flannery et al. 2017); (2) lower self‐organization and problem‐solving scores (Becker et al. 2016); (3) temporal disorganization (Becker et al. 2016); (4) challenges in attentional shifting (Kim and Kim 2021); (5) decreased speed and efficacy of selective attention during initial information processing (Park and Lee 2021); and (6) heightened deficits in self‐regulated learning strategies (Shelton et al. 2019). Research focusing on emotional issues such as stress and social behavior has shown that adults with high CDS scores face a significant risk of displaying suicidal behavior, regardless of other health factors, including symptoms of depression (Becker, Holdaway et al. 2018). Studies have found that higher levels of emotional dysregulation can influence the link between increased CDS scores and social harm, suggesting a strong connection between CDS and social withdrawal (Flannery et al. 2016). In addition, the relationship between CDS scores and sleep patterns indicates that higher CDS scores are associated with greater disruptions in sleep (Fredrick, Yeaman et al. 2022) and poorer sleep quality (Becker et al. 2014).

Research on CDS has advanced significantly, resulting in more efforts to create and test rating scales for evaluating symptoms of this syndrome in children and adolescents, as opposed to adults (Becker 2021). Meanwhile, tests to evaluate characteristics of CDS in adults have been developed and psychometrically validated. Two instruments have been predominantly utilized: the SCT subscale of the Barkley Adult ADHD Rating Scale‐IV (BAARS‐IV) (Barkley 2011) and the Adult Concentration Inventory (ACI) (Becker, Burns et al. 2018; Fredrick, Burns et al. 2022). The BAARS‐IV was the first tool developed to assess the construct validity of self‐reported criteria for diagnosis of CDS in adults (Barkley 2012). In a research involving adults, Barkley (2012) identified nine distinct criteria of SCT that were clearly independent from ADHD symptoms. Further research validated the construct validity of the SCT subscale from the BAARS‐IV in a student demographic (Becker et al. 2014). A study of adults in Japan indicated that only five out of nine items from the SCT subscales of the BAARS‐IV were distinguishable from the inattentive type of ADHD (Takeda et al. 2019), implying potential variability in differentiating CDS from the inattentive type of ADHD in adult populations.

Becker, Burns et al. (2018) recently created the ACI to distinguish symptoms of CDS from the inattentive subtype of ADHD. The preliminary validation of this questionnaire, conducted with a population exceeding 3000 undergraduate students, revealed that just 10 of the 16 items of the ACI were experimentally distinct from symptoms of ADHD and internalizing disorders. Moreover, symptoms of CDS were distinctly linked to compromised emotion control and diminished self‐esteem. Fredrick, Burns et al. (2022) demonstrated that 15 items of the ACI were experimentally different from ADHD symptoms and internalizing disorders.

Research conducted in Turkey demonstrated that the ACI yields a reliable factor structure and acceptable external validity in a clinical adult sample, supporting its applicability beyond Western populations (Yucens et al. 2024). Similarly, Takeda et al. (2019) reported in a Japanese sample of 429 adults (322 university students) that symptoms of CDS were more strongly associated with internalizing symptoms than ADHD‐inattentive symptoms, further underscoring the distinctiveness of the construct across cultural settings. Complementing these findings, an Italian study examining the Child Concentration Inventory–2 in adolescents provided evidence of robust reliability, factor validity, and meaningful neuropsychological correlates in a community sample (Somma et al. 2022). Taken together, these studies indicate that the measurement of CDS demonstrates consistent psychometric properties across diverse populations while also allowing for the identification of culturally specific patterns. Incorporating such comparative perspectives in the current work emphasizes the cross‐cultural robustness of the ACI and strengthens the argument for its use in the Iranian context. Therefore, establishing the construct of CDS in the Iranian context is essential, as it enables more accurate identification of individuals with concentration‐related difficulties who may otherwise remain undiagnosed or misdiagnosed. Given the academic and social challenges associated with CDS, particularly in cultures where scholastic achievement is highly valued, having a culturally validated instrument such as the ACI is crucial for both research and clinical practice. In light of this, the current research investigates the construct validity of the ACI, which is a self‐report measure of CDS in the adult population of Iran.

### 1.2 Current Study

This study evaluated the construct validity of the ACI, a self‐report instrument for CDS, within an adult sample from the Iranian society. Despite an initial validation study of the ACI in college students revealing that 10 of the 16 items exhibited robust factor loadings, the construct validity of CDS may differ among various adult populations (Takeda et al. 2019); therefore, all 16 items were incorporated into the analysis of the current study.

## 2 Methods

### 2.1 Participants and Procedures

The current study involved 2855 individuals aged between 18 and 50 years, who were selected from central and northern cities in Iran. The study included criteria for participant selection to ensure suitability for the research objectives. Participants had to be between the ages of 18 and 50, capable of completing the research questionnaires, and willing to sign an informed consent form. Exclusion criteria were established to maintain the study's integrity; individuals who did not fully complete the questionnaires or reported any physical ailments or psychiatric medication use in the demographic section were excluded, as such issues could potentially skew the research outcomes.

Among the 2855 participants, the average age was 27.42 years. The gender distribution indicated that 69% were female and 31% were male. In terms of employment status, 35% of participants were in permanent employment, 38% were acquiring skills, and 27% were unemployed. This demographic breakdown highlights the diverse backgrounds of participants, which may contribute to the richness of the research findings.

The research employed online data collection methods, which facilitated broader access and encouraged higher participation rates from individuals across different backgrounds. This approach allowed for the inclusion of a more diverse sample, thereby enhancing the representativeness of the data. By reducing geographical and logistical barriers, online administration also minimized participant burden, which may have contributed to more accurate and reliable responses. These factors collectively strengthened the robustness and validity of the findings. In addition, the study was conducted in strict accordance with established ethical guidelines, ensuring informed consent, confidentiality, and the right to withdraw at any stage, thereby safeguarding the rights and well‐being of all participants.

### 2.2 Translation Process

This study had two phases. In Phase 1, the cross‐cultural adaptation process followed Beaton's intercultural debugging criteria (Beaton et al. 2000). This included forward and backward translation, expert consultation, and pilot testing. During the forward translation phase, the item *“My mind feels like it is in a fog”* led to differing interpretations. One translator emphasized a sense of dullness with *“I feel that my mind has become dull and unclear,”* while another highlighted concentration difficulty with *“I have difficulty maintaining attention.”* After thorough discussion, the former translation was selected, as it was judged to align more closely with the intended meaning of the scale.

Phase 2 involved assessing the reliability and validity of the Persian translation of the ACI through a cross‐sectional survey. The initial translation was conducted by two bilingual experts. A linguist and a panel of five Iranian psychologists then synthesized the translations and resolved any discrepancies, producing a preliminary translation titled “Version 1 forward translation Persian‐ACI.”

In the second step of backward translation, an English teacher and a psychologist, both unfamiliar with the original ACI, executed this phase. To assess accuracy, two researchers developed “Version 2.0 backward translation Persian‐ACI,” which was juxtaposed with the original English version of the ACI following the translation of the ACI's initial Persian language version into English.

The pre‐final Version 3.0 of the Persian‐ACI was created with contributions from a panel of two experts tasked with assessing the cultural adaptation of Version 2.0.

### 2.3 Measures

#### 2.3.1 Adult Concentration Inventory

ACI is a self‐report questionnaire designed to assess cognitive insufficiency in adults through 16 questions. Respondents use a four‐point Likert scale, where scores range from 0 (not at all) to 3 (most of the time). Higher scores on the ACI indicate greater difficulty in concentration and cognitive functioning (Fredrick et al. 2020). The ACI has been utilized in various studies, including those by Becker, Burns et al. (2018); Fredrick et al. (2020), specifically among student populations. The tool effectively helps identify challenges related to concentration, making it valuable for both clinical and research applications in understanding cognitive performance and related issues.

#### 2.3.2 Executive Skills Questionnaire‐Revised

The Executive Skills Questionnaire‐Revised (ESQ‐R) was originally crafted by Dawson and Guare (2018) to evaluate executive skills in both adolescents and adults. The ESQ‐R initially comprised 33 questions for adolescents and 36 for adults. Following psychometric revisions by Strait et al. (2020), the questionnaire was streamlined to 25 items that assess five core executive skills: planning, time management, emotion regulation, organization, and behavior regulation. Scoring follows a four‐point Likert scale (0 = never, 3 = always). The Persian version of the ESQ‐R showed strong internal consistency with a total score reliability of 0.91, and specific skills' reliability as follows: planning (0.86), time management (0.62), emotion regulation (0.68), organization (0.72), and behavior regulation (0.79). This indicates that the questionnaire is a reliable measure of executive function skills across different populations.

#### 2.3.3 Patient Health Questionnaire‐9

The Patient Health Questionnaire‐9 (PHQ‐9) is a nine‐item self‐report instrument designed for the purposes of screening, diagnosing, monitoring, and assessing the severity of depression. Respondents answer the questionnaire using a four‐point scale, ranging from 0 to 3, reflecting their circumstances over the preceding 2 weeks. The scoring spectrum extends from 0 to 27, and the completion time is under 5 min. This instrument has undergone rigorous evaluation in clinical settings across various age demographics. The developers employed Cronbach's alpha coefficient to ascertain its internal consistency, which was reported at 0.84, alongside a test‐retest reliability coefficient and a measure of convergent validity, both recorded at 0.84 (Kroenke et al. 2010). In the current study, the internal consistency for the overall score of the PHQ‐9 was determined to be 0.87.

## 3 Results

### 3.1 Descriptive Statistics

Descriptive results showed that participants were in age group of 18–50 years old (M = 27.42 years old) and most of them were female (69%). Furthermore, mean and standard deviation (SD) of variables are considered, and results showed that mean values for research variables were: CDS (M = 12.85, SD = 8.10), depression (M = 8.61, SD = 5.83), and executive functions (M = 26.51, SD = 12.18). Before we could validate this scale, its prerequisites, including data normal distribution, were considered using Shapiro–Wilk test, and results showed that data distribution for all variables was non‐normal (*p* < 0.05).

### 3.2 Structural Validity

To test structural validity, we made use of explanatory factor analysis (EFA). To this end and before implementing factor analysis, sampling adequacy using Kaiser–Meyer–Olkin (KMO) test and Bartlett's test of sphericity was tested, and results showed that the application of factor analysis for our data was suitable (KMO = 0.916, *X* ^2^ = 6910.582, df = 120, *p* < 0.001). Then, we test the correlation among scale items with the whole scale, and results showed that the correlation of all items with the whole scale was higher than 0.37, suggesting the high correlation among items and the whole scale and therefore it was suitable for factor analysis. In the third section, we should extract the primary factors. To this end, we made use of correlation matrix and principal component analysis due to the fact that our objective is to explain the total variance of correlation matrix. We made use of the scree test to determine that how many factors there are to rotate (see Table 1). Three factors were extracted totally and the eigenvalues and explained variance percent for each of the factors before and after rotation showed that the first principal component has the highest eigenvalue (3.813) and it explains the highest variance. Results showed that the sum of explained variance is 49.56%, of which 34.443% is dedicated to the first factor.

**TABLE 1:** Rotated component matrix.

	1	2	3
S15	0.723		
S14	0.711		
S13	0.702		
S11	0.651		
S16	0.639		
S10	0.598		
S6	0.549		
S12	0.506		
S2	0.407		
S7		0.704	
S1		0.645	
S9		0.615	
S4		0.526	
S5			0.456
S8			0.553
S3			0.376

In next stage, we consider the factor loadings of items and the rotated matrix with Kaiser Normalization method that converged in 50 iterations. Results showed that three factors have the highest eigenvalues that the factor loadings and factor structure confirmed it (see Table 2). Therefore, the given ACI scale has three factors, which the scree test also confirmed these results.

**TABLE 2:** Results of structural validity.

		Items	Factor loading	*t* value	Cronbach's alpha	CR	AVR
SCT	Attention problems	S15	0.638	34.523	0.851	0.884	0.561
		S14	0.760	57.871			
		S13	0.776	69.457			
		S11	0.767	57.297			
		S16	0.586	30.092			
		S10	0.572	26.153			
		S6	0.679	40.058			
		S12	0.684	45.316			
		S2	0.612	30.501			
	Slow	S1	0.493	14.627	0.768	0.798	0.559
		S4	0.677	35.436			
		S7	0.729	45.364			
		S9	0.776	57.420			
	Imagination	S3	0.718	37.944	0.723	0.752	0.509
		S5	0.561	19.463			
		S8	0.835	91.114			

### 3.3 Internal Consistency

To consider the internal validity of the scale, we made use of Cronbach's alpha, and results showed that its internal consistency is acceptable (*α* = 0.864). The Cronbach's alpha if‐item‐deleted coefficients ranged from 0.84 to 0.87. The correlated item‐total correlations indicated that all items were significantly correlated with total ACI scores. Furthermore, to test the consistency of the scale items, we made use of correlation of each item with the total score of the scale, and results showed that the correlation of all items with the total score of the scale was higher than 0.54, suggesting that the items of the scale have acceptable internal consistency. These values support the internal consistency of the ACI scale for the Iranian population.

### 3.4 Confirmatory Factor Analysis

To confirm structural validity, confirmatory factor analysis (CFA) was run on the samples using Smart‐Plas‐3. According to EFA results, our hypothetical factor model contained three factors: (1) attention problems, (2) sluggishness, and (3) mind‐wandering. Based on the fit indices, the original correlated three‐factor model of the ACI was considered adequate: *χ* ^2^ = 815.20; df = 101; RMSEA = 0.050; CFI = 0.95; TLI = 0.94; IFI = 0.95; SRMR = 0.03.

**FIGURE 1:**
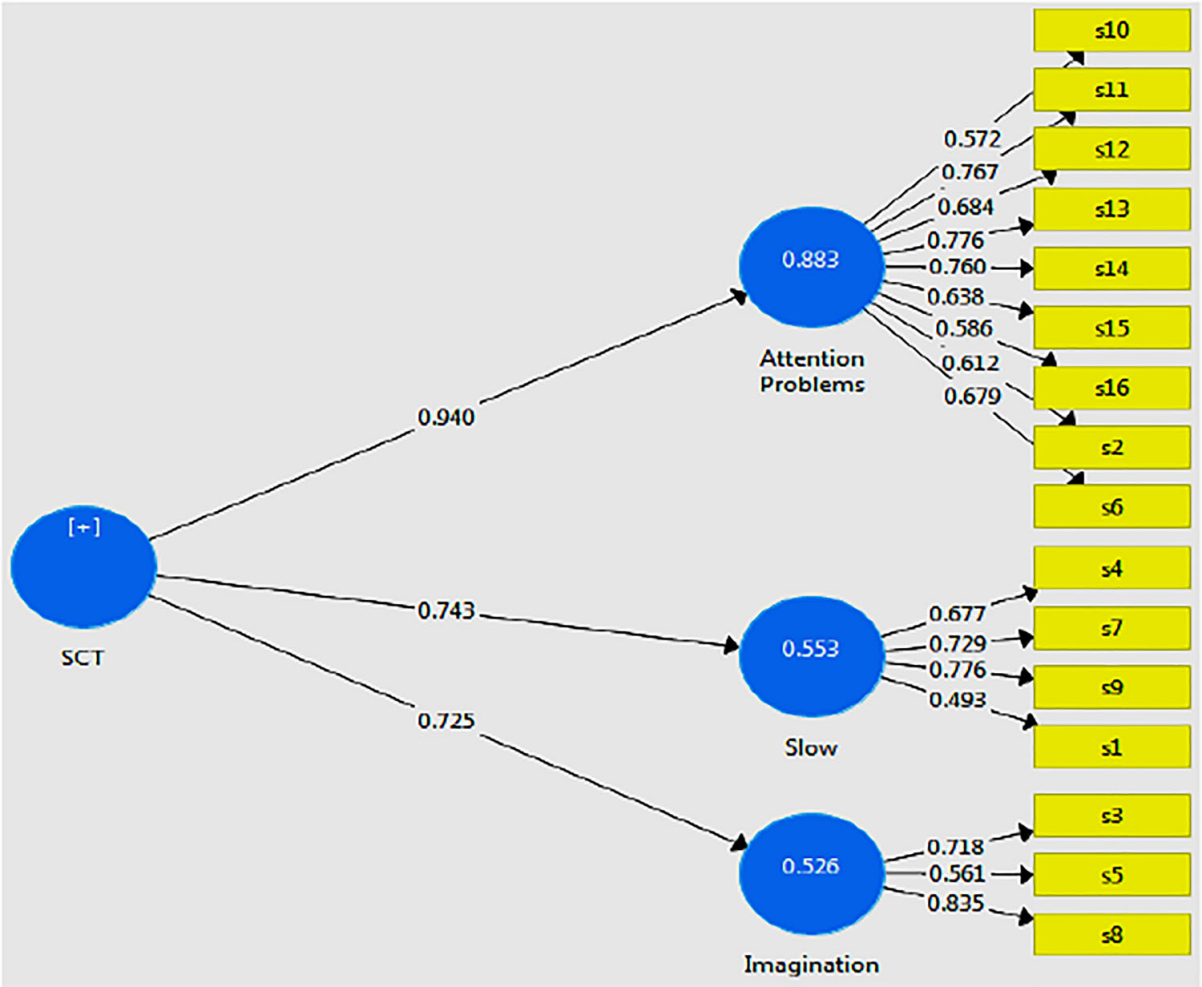
The tested model of the research (based on path coefficients).

**FIGURE 2:**
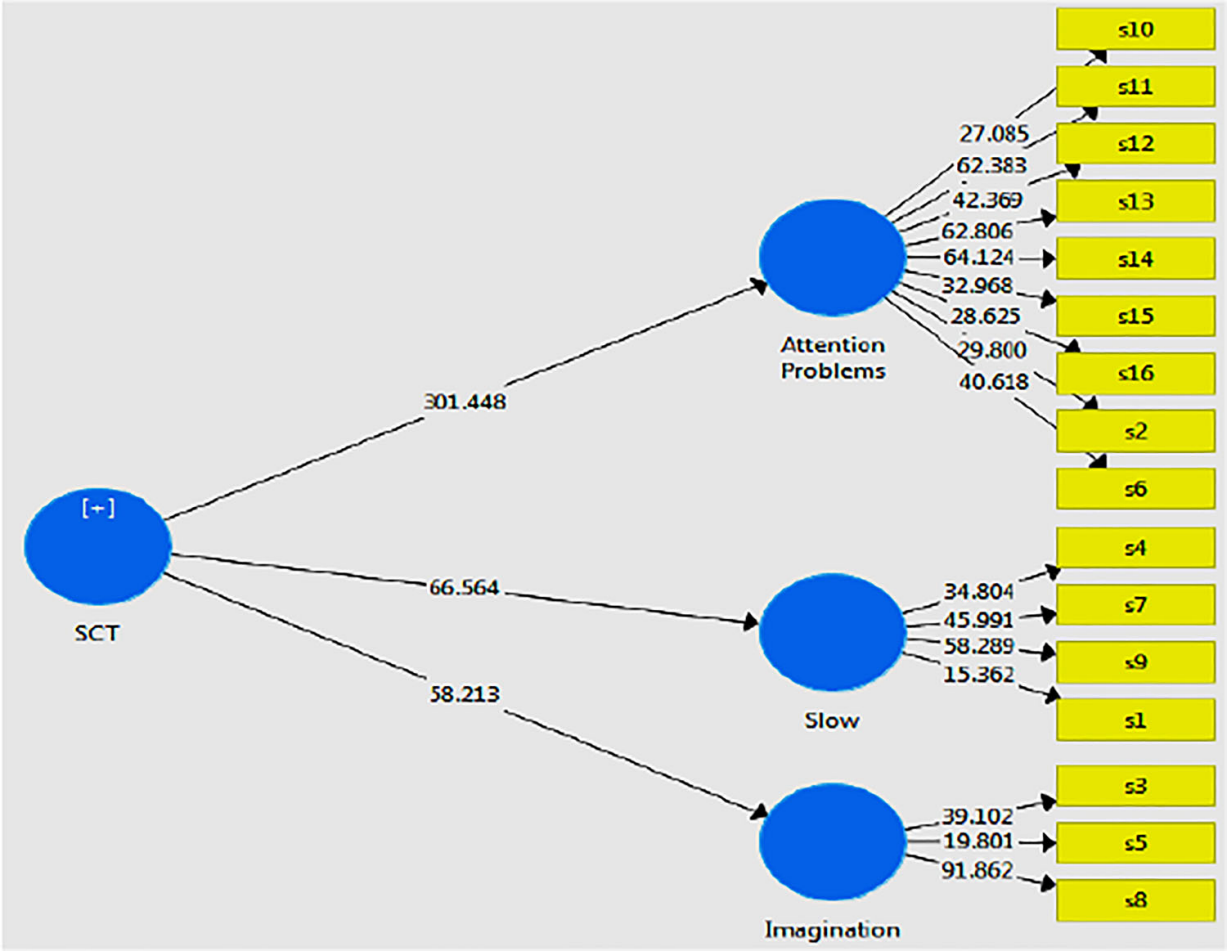
The tested model of the research (based on the *t* value).

The structural model of the research and corresponding path coefficients are presented in (Figure 1), and the t‐value model is shown in (Figure 2). Based on the research model, factor loadings, *t* value, Cronbach's alpha, composite reliability (CR), and average variance extracted (AVR) (Table 3), results showed that factor loadings for all items were higher than 0.4 and therefore we could say with confidence that factor loadings of all items are suitable. Furthermore, *t* value for all items was higher than 1.96 and therefore all items were significant at 0.01% (*p* < 0.01). Besides, Cronbach's alpha for three factors of the ACI scale for the Iranian population was higher than 0.7 (attention problems = 0.851, sluggishness = 0.768, and mind‐wandering = 0.723) and therefore we can state with confidence that its reliability is suitable. Next, CR results showed that it was higher than 0.7 for three factors of the ACI scale for the Iranian population (attention problems = 0.884, sluggishness = 0.798, and mind‐wandering = 0.752) and therefore they have good internal consistency. Finally, the AVE values for all three factors (attention problems = 0.561, sluggishness = 0.559, and mind‐wandering = 0.509) exceeded the 0.5 threshold, confirming the convergent validity and structural reliability of the ACI for the Iranian population.

**TABLE 3:** Discriminant validity.

	1	2	3
1. Attention problems	0.679		
2. Imagination	0.566	0.713	
3. Slow	0.548	0.408	0.677

### 3.5 Divergent Validity

Divergent validity measures the ability of a measurement model to differentiate the observables of the hidden variable of that model with other observables in the model, and is actually a complement to convergent validity, which is measured through the Fornell–Larcker test. Results of the divergent validity test showed that the root value of the AVE variables in the current study, which are located in the boxes in the main diagonal of the matrix, is higher than the correlation value between them, which are arranged in the lower and left boxes of the main diameter. Therefore, it can be stated that the constructs (implying variables) in the model interact more with their items than with other constructs.

Furthermore, structural fit indices include determination coefficient or *R* ^2^ and *Q* ^2^ criterion. *R* ^2^ value higher than 0.33 shows the strength of the relationship between that construct and endogenous constructs (Henseler et al. 2009). The *Q* ^2^ value for all endogenous constructs determines three values of 0.02, 0.15, and 0.35 as low, medium, and strong predictive power (Stone–Geisser). Results showed that *Q* ^2^ value for three subscales of study was higher than 0.32 (attention problems = 0.330, sluggishness = 0.327, and mind‐wandering = 0.381), suggested the ability to predict indicators related to the endogenous structures of the model. Furthermore, the *R* ^2^ values for the three subscales (attention problems = 0.403, sluggishness = 0.451, and mind‐wandering = 0.462) exceeded the 0.33 threshold, suggesting that the relationships among endogenous constructs in the research model were strong. Therefore, we could trust to model fit structurally. Finally and to check the fit of the overall model, there is only one criterion called GOF with the formula of $\mathrm{GOF}=\sqrt{\bar{\mathrm{Communalities}}\times \bar{R^{2}}}$, in which three values of 0.01, 0.25, and 0.36 are introduced as weak, medium, and strong values for GOF (Tenenhaus et al. 2004). The GOF value was 0.390, which could be considered strong. Therefore, based on the mentioned classification, it shows the good fit of the overall research model.

### 3.6 Concurrent Validity

To validate ACI scale from concurrent validity view, we calculate the correlation between ACI, depression, and executive functions using Spearman correlation coefficient.

#### 3.6.1 Relationship Between ACI and Executive Functions

Results showed that there was a strong significant relationship between executive functions and three subscales of ACI, including attention problems (*r* = 0.711, *p* < 0.01), sluggishness (*r* = 0.670, *p* < 0.01), and mind‐wandering (*r* = 0.537, *p* < 0.01), and between the whole scale and executive functions (*r* = 0.743, *p* < 0.01).

#### 3.6.2 Relationship Between ACI and Depression

Results showed that there was a significant relationship between depression and three subscales of ACI, including attention problem (r = 0.690, P<0.01), sluggishness (r = 0.599, P<0.01), and mind‐wandering (r = 0.504, P<0.01), and between the whole scale and depression (r = 0.703, P<0.01).

## 4 Discussion

The objective of this study was to examine the psychometric features of the ACI in assessing CDS in non‐English speaking population. The study assessed the structural and external validity of the ACI questionnaire, along with its validity and reliability, and evaluated its concurrent validity in relation to the depression and executive function.

EFA was employed to assess the structural validity of all 16 factors of the questionnaire. The findings indicated that three factors possessed the highest eigenvalues, as corroborated by the scree test. The CFA demonstrated that the three proposed factors (attention problems, sluggishness, and mind‐wandering) were validated and exhibited a satisfactory match. In the subsequent phase, to assess the convergent validity of the ACI, measures of depression and executive function deficits were employed, yielding results that demonstrated adequate convergent validity of the questionnaire. The Fornell–Larcker test was employed to assess divergent validity, and the findings confirmed its appropriateness. The reliability of the questionnaire was assessed using Cronbach's alpha method, which demonstrated its suitability. The correlation test results indicate a significant positive relationship between ACI and depression. Previous research has shown a strong and direct association between various types of SCT and depression (Swope et al. 2020). In addition, it has been noted that there is a conceptual overlap in the traits associated with SCT and depression (Smith et al. 2020). However, distinct symptoms differentiate the two conditions: CDS is characterized by excessive daydreaming, diminished concentration, and a tendency to stare, while depression features suicidal thoughts, hopelessness, overwhelming guilt, and feelings of worthlessness (Smith et al. 2020). Research suggests that rumination and disorganized thinking in individuals with CDS may lead to the onset of depressive disorders (Becker, Webb et al. 2021). Miller et al. (2024) also argued that factors associated with CDS, such as social isolation, loneliness, and low self‐esteem, could contribute to the development of depression (Ergül and Ersöz Alan 2024) over time in affected individuals. It is important to note that some studies have suggested that individuals with depression might use mind‐wandering as a coping mechanism to deal with their problems, challenging mental states, and monotonous lives (Becker and Barkley 2021). While the current study highlights a correlation between CDS and depression, further empirical research is necessary to determine the directionality of this relationship.

The current study's correlation test results reveal a substantial positive correlation of 0.743 between CDS and executive functions; prior research indicates that CDS symptoms are linked to executive function dysfunction such as self‐organization, planning, problem‐solving, and emotional and behavioral regulation in individuals with ADHD (Barkley 2018). Leikauf and Solanto (2017) demonstrated a substantial correlation between CDS and time management. Issues in time management linked to CDS may relate to the slowness explicitly indicated in the disorder's nomenclature (slowness of thinking and behavior) (Tirapu‐Ustárroz et al. 2015), prompting some researchers to attribute the disorder's etiology to an arousal imbalance. Time management and self‐organization or problem‐solving difficulties in this disease may be attributed to issues with processing speed and latency (Yung et al. 2020). The findings of Araujo Jiménez et al. (2015) indicated that the metacognitive components of executive functions (planning/organization, monitoring, and task initiation) are associated with cognitive impairment. Barkley (2013) discovered in his research that executive functions (inhibition, working memory, and planning and organizing) are among the most significant predictors of cognitive impairment. Becker and Barkley (2021) assert that inhibition in CDS may be inherently cognitive, resulting in a particular propensity for individuals to engage in daydreaming, rumination, and mental disarray. Tamm et al. (2018) demonstrated that CDS can result in deficiencies in visual and perceptual skills, spatial abilities, attention to detail, and processing speed, all of which are essential aspects of executive functioning.

Validating the ACI for use in the Iranian population carries several important clinical implications. First, it provides clinicians and mental health professionals with a culturally adapted, psychometrically sound tool for identifying symptoms of CDS, a construct increasingly recognized as distinct from ADHD. Having a reliable measure enables more accurate differential diagnosis, reducing the risk of misdiagnosis and inappropriate treatment. Second, the availability of the Persian‐ACI allows for early detection of attentional disengagement, mental fogginess, and related symptoms in clinical and educational settings. This facilitates timely intervention, which can improve academic performance, emotional well‐being, and daily functioning in individuals affected by CDS. Third, the validated instrument supports treatment planning and monitoring by providing a standardized method to track symptom severity and treatment response over time. This may guide the tailoring of psychological interventions, such as cognitive‐behavioral strategies or attention‐enhancement programs, to the needs of Iranian patients.

In spite of the strengths and clinical implications of this study, several limitations should be acknowledged. First, the study relied exclusively on self‐report measures, which may be subject to response bias and may not fully capture the complexity of CDS. Second, clinical diagnostic confirmation was not included, which limits the ability to directly relate ACI scores to formal psychiatric diagnoses. Third, the use of a cross‐sectional design restricts causal inferences; future longitudinal studies would help clarify the stability and predictive validity of the findings. Fourth, the sampling method may limit the representativeness of the results, and the absence of socioeconomic data prevents examination of how social or economic factors might influence CDS symptoms. Finally, the lack of cross‐cultural comparisons reduces the ability to contextualize these findings within broader international research. Despite these constraints, the present study makes an important contribution by validating the ACI in the Iranian context and providing a foundation for future research (Figure 1) (Figure 2).

## Author Contributions

**Karim Abdolmohamadi**: project administration, writing – original draft, supervision, conceptualization. **Asgar Alimohamadi**: formal analysis, writing – review and editing, methodology.

## Funding

The authors have nothing to report.

## Ethics Statement

The procedures of this study were conducted in accordance with the principles of the Declaration of Helsinki.

## Consent

Informed consent was received from all participants.

## Conflicts of Interest

The authors declare no conflicts of interest.

5

## Data Availability

The data that support the findings of this study are available on request from the corresponding author. The data are not publicly available due to privacy or ethical restrictions.
